# Integrated Clinomics and Molecular Dynamics Simulation Approaches Reveal the SAA1.1 Allele as a Biomarker in Alkaptonuria Disease Severity

**DOI:** 10.3390/biom15020194

**Published:** 2025-01-29

**Authors:** Alfonso Trezza, Bianca Roncaglia, Anna Visibelli, Roberta Barletta, Luana Peruzzi, Barbara Marzocchi, Daniela Braconi, Ottavia Spiga, Annalisa Santucci

**Affiliations:** 1 ONE-HEALTH Laboratory, Department of Biotechnology, Chemistry and Pharmacy, University of Siena, Via Aldo Moro, 53100 Siena, Italy; alfonso.trezza2@unisi.it (A.T.); bianca.roncaglia@unisi.it (B.R.); anna.visibelli2@unisi.it (A.V.); r.barletta@student.unisi.it (R.B.); luana.peruzzi@unisi.it (L.P.); barbara.marzocchi@unisi.it (B.M.); daniela.braconi@unisi.it (D.B.); ottavia.spiga@unisi.it (O.S.); 2 MetabERN, Department of Biotechnology, Chemistry and Pharmacy, University of Siena, Via Aldo Moro, 53100 Siena, Italy

**Keywords:** AKU, clinomics, biomarker, SAA1.1, molecular dynamics simulation, molecular modeling, amyloidosis, omics, data integration

## Abstract

Alkaptonuria (AKU) is a rare metabolic disorder characterized by the accumulation of homogentisic acid (HGA), leading to progressive ochronosis and joint degeneration. While much is known about HGA’s role in tissue damage, the molecular mechanisms underlying acute inflammation in AKU remain poorly understood. Serum amyloid A (SAA) proteins are key mediators of the inflammatory response, yet their potential as biomarkers for inflammation in AKU has not been explored. This study investigated the role of the SAA1.1 allele as a biomarker for the severity of acute inflammation in AKU. Data from the ApreciseKUre Precision Medicine Ecosystem were analyzed to assess the relationship between SAA1 allelic variants and inflammatory markers. Molecular dynamics simulations compared the structural dynamics of SAA1.1 and SAA1.2 isoforms, with standard modeling and analysis pipelines employed. Using a clinomics approach, we showed that AKU patients expressing the SAA1.1 allele have significantly higher acute inflammation-related markers. Extensive molecular dynamics simulations revealed that the SAA1.1 isoform lent high structural instability of the C-terminal domain, accelerating the formation of amyloid fibrils and exacerbating the inflammatory condition. These findings would identify the SAA1.1 allele as a novel genetic biomarker for the progression of secondary amyloidosis in AKU and its severity. Furthermore, new molecular insights into the inflammatory mechanisms of AKU were provided, suggesting potential therapeutic approaches aimed at stabilizing SAA1.1 protein and preventing amyloid fibril formation, with significant implications in AKU and precision medicine strategies for SAA-related diseases.

## 1. Introduction

Alkaptonuria (AKU) is a rare autosomal recessive metabolic disorder (OMIM 203500, prevalence <0.1 per 10,000 individuals) that results in early-onset and progressively debilitating spondyloarthropathy [1]. Caused by mutations in the homogentisate 1,2-dioxygenase (HGD) gene, AKU disrupts tyrosine and phenylalanine metabolism [2]. This disruption causes an abnormal homogentisic acid (HGA) build-up, a metabolic intermediate that, upon accumulation, leads to ochronosis. This dark, insoluble pigment deposits within connective tissues, especially cartilage and bone. These ochronotic deposits cause tissue hardening and pigmentation, which contribute to AKU’s hallmark chronic inflammation, joint deterioration, and multiorgan complications, including cardiac and renal involvement [3].

Over time, AKU patients experience severe and disabling pain and a marked reduction in quality of life due to these cumulative pathological effects.

SAA is a family of acute-phase proteins synthesized by hepatocytes in response to inflammatory stimuli. In humans, four SAA genes (SAA1, SAA2, SAA3, and SAA4), located within a 150 kb region on chromosome 11p15.1, encode SAA proteins with substantial sequence similarity [4,5,6]. Under normal conditions, the plasma levels of SAA are approximately 3 mg/L. Still, they can surge to over 2000 mg/L during inflammation, primarily due to the action of pro-inflammatory cytokines such as IL-1, IL-6, and TNF-α, which enhance SAA gene expression [7,8]. A distinctive aspect of SAA lies in its role in amyloidosis, particularly AA amyloidosis, a condition characterized by the extracellular accumulation of insoluble protein fibrils that damage tissue and organ function [9,10]. Prolonged inflammation, as seen in AKU, can trigger AA amyloidosis: SAA undergoes aberrant cleavage, misfolding, and aggregation, adopting a pathological β-sheet conformation that promotes amyloid fibril formation [11,12].

Among SAA proteins, SAA1 has received significant attention due to its role in inflammatory responses and its genetic polymorphisms [13]. The SAA1 gene encodes a precursor protein of 122 amino acids, including an 18-amino acid signal peptide. SAA1 is built up from helices 1, 2, 3, and 4, comprising residues 1–27, 32–47, 50–69, and 73–88, respectively. Unlike conventional helix bundles, the SAA1 helix bundle adopts a cone-shaped topology, with the N-termini of helices 1 and 3 closely packing against the C-termini of helices 2 and 4 [14]. The C-terminal tail, encompassing residues 89–104, is well ordered and wraps around one face of the helix bundle, stabilizing it through multiple salt bridges and hydrogen bonds with residues from helices 1, 2, and 4. These interactions include Glu-26 and Tyr-29 from helix 1, Tyr-35 and Arg-39 from helix 2, and Trp-85 and Gly-86 from helix 4 [14]. A critical structural feature is the invariant charge cluster formed by Tyr-104, Tyr-35, Arg-39, and Arg-96, where bifurcated salt bridges involving the carboxylate termini and the hydroxyl group of Tyr-35 stabilize the C-terminal region. This charge quartet is conserved across SAA proteins in all species. Additionally, the C-terminal tail contains three invariant proline residues at positions 92, 97, and 101, which impose further conformational rigidity. Collectively, these conserved interactions emphasize the role of the C-terminal tail in maintaining the structural integrity of the SAA1 helix bundle [14].

Following cleavage of its signal peptide, a mature 104-amino-acid SAA1 protein is released into the bloodstream, where it participates in various physiological processes [12]. Currently, five known SAA1 polymorphisms (SAA1.1, SAA1.2, SAA1.3, SAA1.4, and SAA1.5) are reported, with 274 variants annotated on the UniProt Database, including missense mutations along the entire protein sequence, each with varying clinical significance [13]. Specifically, the N-terminal and C-terminal regions of SAA1 work together to regulate amyloid formation. The C-terminal tail stabilizes the protein’s structure by wrapping around its four-helix bundle and locking it into a non-amyloidogenic hexamer. The N-terminal region, containing amyloid-prone segments, is normally hidden within this structure, preventing fibril formation. However, under inflammatory conditions, glycosaminoglycans (GAGs) like heparan sulfate can displace high-density lipoprotein (HDL) from SAA, exposing the N-terminal and promoting aggregation. Additionally, SAA1.1 is more susceptible to MMP-1-mediated degradation than other SAA1 variants, leading to the generation of amyloidogenic N-terminal fragments, such as the 1–57 fragment, which further enhance fibril formation in pathological states [14]. Consistently, previous work [15] showed that SAA1.1 was the major isoform present in the amyloid deposits of all affected individuals, including those heterozygous for SAA1.1. These findings highlight the critical role of isoform-specific structural properties and susceptibilities in amyloidogenesis [15]. Thus, the C-terminal maintains stability, while the N-terminal holds amyloid-forming potential that is activated in pathological states [15].

Recent studies have highlighted the role of serum amyloid A (SAA) proteins in the pathophysiology of AKU, specifically associating the disease with secondary amyloid A (AA) amyloidosis [16].

Furthermore, in AKU, the presence of elevated HGA appears to increase the misfolding of SAA1, acting as a destabilizing factor that accelerates the transition of SAA1 proteins to amyloidogenic conformations [17]. This biochemical interplay between HGA and SAA1 aggravates amyloid deposition in affected tissues, potentially worsening inflammation and organ damage. Notably, previous works highlighted the propensity of the SAA1.1 polymorphism, distinguished from other variants by the presence of the Ala52Val, Val57Ala, and Thr59Ser mutations, to induce an early and more severe inflammatory response compared to the other SAA1 variants [18].

Despite the known role of SAA proteins in amyloidosis, the specific involvement of the SAA1.1 isoform in alkaptonuria (AKU) has not been previously established [11,12,13,14,15,16,17]. While these studies have explored the role of SAA1 and its variants in amyloidogenesis, they have not specifically linked the SAA1.1 isoform to AKU severity. This study aims to fill this gap by identifying SAA1.1 as a potential biomarker for AKU severity and progression. Our research design differs from previous studies by integrating a comparative analysis using the ApreciseKUre Precision Medicine Ecosystem (AKU-PME) [19]. This study not only analyzes the relationship between SAA1.1 and markers of inflammation but also investigates the underlying structural mechanisms of SAA1.1’s amyloidogenic properties using molecular dynamics simulations. Our findings indicate that SAA1.1 exhibits significant structural instability, particularly in the C-terminal region, which enhances its tendency to form amyloid fibrils. This structural destabilization is consistent with our clinomics analysis, which highlights the potential of SAA1.1 as a high-risk biomarker for predicting the severity of amyloid-related complications in AKU.

The ApreciseKUre Precision Medicine Ecosystem (AKU-PME) platform is a comprehensive, multidisciplinary, and interactive database that integrates genetic, biochemical, and clinical data on AKU [19]. This integrative study, utilizing data from AKU-PME, investigated the relationships between SAA variants and markers of inflammation and metabolism. To uncover the molecular features that grant SAA1.1—identified as the predominant isoform in amyloid deposits—its strong amyloidogenic properties, a comparative analysis was performed. Molecular dynamics simulations were used to compare SAA1.1 with the reference sequence from the UniProt database, corresponding to SAA1.2, which reflects the translation of the current human genome reference assembly GRCh38/hg38. Our analyses confirmed the clinomics approach, shedding light on a lower C-ter region structural stability of SAA1.1, reflecting an SAA1.1 early structural destabilization and its significant increase in the fibrilization process compared to the other variant. Our findings support the presence of the SAA1.1 allelic variant as a high-risk biomarker for predicting a more severe and early-onset amyloidogenic profile in AKU, paving the way for more personalized therapeutic strategies targeting inflammation management in patients with an elevated risk of amyloid complications. Beyond AKU, these insights may also broaden our understanding of the role of SAA polymorphisms in other inflammatory conditions associated with secondary amyloidosis.

## 2. Materials and Methods

### 2.1. ApreciseKUre Digital Ecosystem

The ApreciseKUre Precision Medicine Ecosystem (PME) platform is a comprehensive, multidisciplinary, and interactive database (available at www.bio.unisi.it/aprecisekure/) (accessed on 20 November 2024) [19].

It integrates genetic, biochemical, and clinical data on alkaptonuria (AKU), aiming to address the challenges of conducting in-depth studies and developing effective treatment strategies for this rare disease. The ApreciseKUre ecosystem facilitates the collection, integration, and analysis of AKU-related data from diverse research groups. It harmonizes and standardizes varied datasets, creating a global, searchable repository for AKU that serves as a crucial reference point. This ecosystem not only enables the organization of data from multiple sources but also enhances accessibility and usability for clinicians and researchers by standardizing the information. Through computational modeling and database management, ApreciseKUre provides a comprehensive and dynamic view of the AKU patient journey. By integrating data layers, such as patient-reported outcomes, physician assessments, test results, genotypic data, and molecular modeling of proteins, it supports clinicians and researchers in identifying potential biomarkers for patient stratification and personalizing treatment strategies [20]. This unified platform ultimately aims to enhance AKU’s precision medicine approach, driving research and clinical care forward with robust, accessible data [21].

### 2.2. Comparative Analysis of SAA1 Gene Variants and Plasma Biochemical Metrics

In this study, we performed a preliminary analysis to investigate the relationship between SAA1 polymorphisms expressed in AKU patients and various plasma biochemical metrics obtained from the ApreciseKUre PME. Data processing was conducted using Python (version 3.10), with the Pandas library (version 1.5.3) employed for data manipulation and organization. We examined these genetic variants in the context of metrics related to inflammation and metabolism, including biomarkers of oxidative stress, inflammation, and disease progression, such as SAA, creatinine levels, plasma amino acids, hypoxanthine, xanthine, and uridine. To effectively visualize and compare the distribution of each biochemical metric across the two gene groups, we used a violin plot. This plot combines information on each metric’s distribution shape and density, allowing us to detect differences in dispersion between the groups.

### 2.3. Structural Resource, MD Simulations, and Conformational Analyses

The primary structure of the human SAA1.1 was retrieved from the COSMIC variant with code COSM4590767 [22]. In contrast, SAA1.2 (translation of the current human genome reference assembly GRCh38/hg38) was retrieved from the UniProtKB reviewed (Swiss-Prot) database (code P0DJI8) [23]. The human apo-state Serum Amyloid A1 3D structure (SAA1.1), obtained using an X-ray method with a resolution of 2.19 Å (PDB ID: 4IP8), was downloaded from the RCSB PDB database [11]. To avoid errors during the molecular dynamic (MD) simulations, potential 3D structure-missing amino acid side chains were added using PyMOD3.0 (Department of Biochemical Sciences, Sapienza University, Rome, Italy) [24] and validated through PROCHECK [25]. In brief, both SAA1.1 and SAA1.2 primary structures (in fasta format) and 3D structures (in pdb format) were downloaded and uploaded on PyMOD3.0, so the primary structures (target sequence) were aligned with the primary structure of the corresponding pdb structure (template sequence) using CLUSTAL Omega as an alignment tool, and then the MODELLER tool performed the optimization of the 3D structures. The structures were saved in pdb format and their potential steric clashes were solved using energy minimization, and the structure was relaxed using a short MD run of 50 ns. Thus, the CHARMM-GUI platform (Lehigh University, Bethlehem, PA, USA) [26] was used to assign all molecular parameters for the cMD using the charmm36-mar2019 force field, while GROMACS 2019.3 was used for further analysis (Department of Biophysical Chemistry, Groningen University, Groningen, The Netherlands) [27]. The structure was put in a cubic box, TIP3P water molecules were added, and then the system was neutralized with counter ions. The energy minimization was performed in 5000 steps using the steepest descent algorithm (forces less than 100 kJ/mol/nm). The integration of the MD was performed at each time step of 2 fs; a V-rescale thermostat and the Nose–Hoover barostat kept the temperature and pressure at 300 K and 1 atm, respectively. The MD run lasted 100 ns to relax the system. This 3D structure was used as the starting structure to obtain the SAA1.2 3D structure, using DUET tool [28] to carry out the Ala52Val, Val57Ala, and Thr59Ser mutations. Then, the obtained 3D structure was processed as previously described for SAA1.1. Both structures underwent an MD run of 1 µs (1000 ns). All MD analyses were performed with the GROMACS 2019.3 package [29]; in detail, RMSD and RMSF analyses were evaluated with “gmx rms” and “gmx rmsf (using the flag -res)” function, respectively, implemented in GROMACS 2019.3, while the “gmx mdmat” function was used to evaluate the distance matrices consisting of the smallest distance between residue pairs; all parameters were used by default. The Bio3D library implemented in R studio (Posit PBC, Wien, Austria) [30] analyzed the MD trajectory (in xtc format) to provide the system conformational distribution, the principal component analysis (PCA), and the intramolecular interaction network, using all parameters by default. GRACE generated the MD graphs and PyMOL 3.3 was used as a molecular graphic interface, produced the biological system pictures/videos, and visualized the MD trajectories.

## 3. Results

### 3.1. Comparative Analysis of SAA1.1 in ApreciseKUre PME

**Figure 1:**
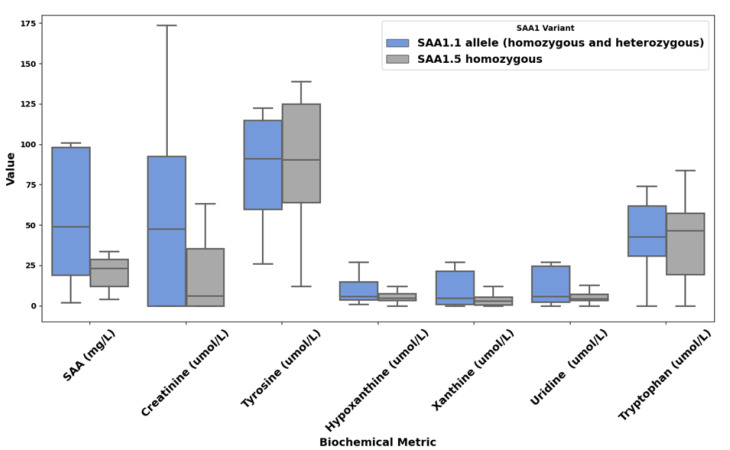
Boxplot comparison of biochemical metrics across SAA1 variants. The blue boxes represent individuals with the SAA1.1 allele (homozygous and heterozygous), while the gray boxes denote individuals homozygous for SAA1.5. Median values, interquartile ranges, and variability are displayed for each biochemical parameter.

In the ApreciseKUre PME, the expression of three allelic polymorphisms of SAA1 (SAA1.1, SAA1.3, and SAA1.5) was considered. Among these, SAA1.3 was poorly represented and detected only in heterozygosity, whereas SAA1.1 and SAA1.5 were more prevalent. The comparative analysis of plasma biochemical metrics revealed significant differences between individuals characterized by the presence of the SAA1.1 allele, whether in homo- or heterozygosity (1.1/1.1, 1.1/1.3, and 1.1/1.5), and the SAA1.5/1.5 homozygotes. These differences spanned several markers of inflammation and metabolism, as visualized in Figure 1.

Among the key inflammatory markers, serum amyloid A (SAA) levels were significantly elevated in the SAA1.1 group, with a mean of 72.85 mg/L compared to 27.26 mg/L in SAA1.5 homozygotes. This finding highlights the enhanced systemic inflammatory response associated with the SAA1.1 allele. Metabolic markers, including creatinine and tryptophan, also differed between the groups. Tyrosine levels showed slightly lower mean values in the SAA1.1 group (112.20 µmol/L) compared to SAA1.5/1.5 individuals (120.46 µmol/L), suggesting consistent disruptions in tyrosine metabolism across all AKU patients, regardless of the allele. Tryptophan levels were similar between groups, with mean values of 50.59 µmol/L and 51.83 µmol/L in SAA1.1 and SAA1.5/1.5 individuals, respectively, indicating that tryptophan metabolism is similarly affected in both allelic profiles. Creatinine levels were elevated in the SAA1.1 group, with a mean value of 125.1 µmol/L compared to 107.4 µmol/L in the SAA1.5/1.5 group, suggesting potential renal involvement or altered metabolic regulation. Xanthine and hypoxanthine levels displayed a marked increase in the SAA1.1 group, with a mean of 6.62 µmol/L and 7.18 µmol/L compared to 2.27 µmol/L and 3.29 µmol/L in the SAA1.5/1.5 group, respectively. This striking difference hints at a significant alteration in purine metabolism associated with the SAA1.1 allele, potentially contributing to AKU’s inflammatory and amyloidogenic processes. These preliminary findings underscore the impact of SAA1 polymorphisms on AKU’s metabolic and inflammatory landscape. The elevated SAA levels in the SAA1.1 group reflect a heightened susceptibility to amyloidogenesis and inflammation, consistent with the established predisposition of the SAA1.1 allele to more severe amyloid complications. Once the predisposition of the SAA1.1 allele to higher disease severity was confirmed, further investigation into the structural stability and molecular dynamics of the SAA1.1 polymorphism was carried out.

### 3.2. cMD: Structural Stability and Protein Flexibility

**Figure 2:**
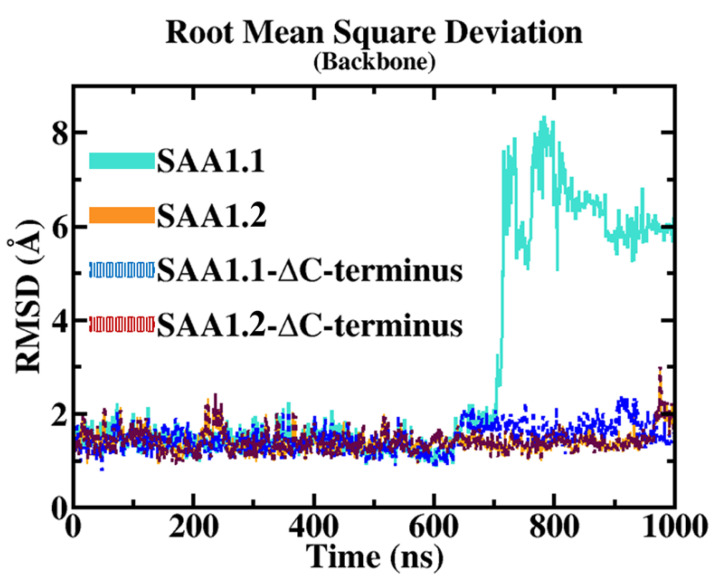
RMSD trend.

To explore molecular insights of the SAA1 variant structural conformations, a cMD run of 1 µs was performed for each biological system. The target backbone structural stability was computed through RMSD analyses to avoid potential computational bias. The RMSD results showed high structural integrity for SAA1.2, exhibiting a stable trend along the entire MD run. Contrarily, the SAA1.1 mutant showed a stable trend for the first 700 ns, then the RMSD values rapidly increased to 6 Å until the end of the MD run (Figure 2). To further explore structural insights, the stability of the protein backbones not considering the C-ter region was examined. From the RMSD results, the SAA1.1 and SAA1.2 backbone were highly stable with an RMSD value of 1.5 Å during the MD run, suggesting that the C-ter region in SAA1.1 might be involved in SAA1.1 structural destabilizing (Figure 2).

**Figure 3:**
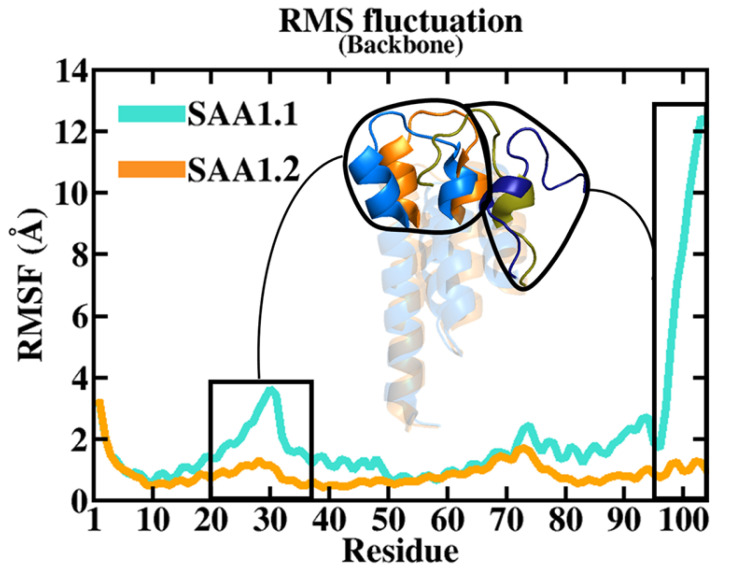
RMSF profile. The RMSF trends of SAA1.1 and SAA1.2 are reported in light blue and light orange lines, respectively. The black box encloses the alpha–alpha hairpin region (20–37) and C-ter domain (97–104). The enlargement represents the alpha–alpha hairpin region (blue for SAA1.1 and orange for SAA1.2) and C-ter domain (dark blue for SAA1.1 and golden for SAA1.2) of SAA1 variants. The higher fluctuation and displacement of the SAA1.1 alpha–alpha hairpin region from the C-ter domain is evident compared to the SAA1.2.

To further investigate the role of the C-ter region in the stability of the SAA1 variants, RMSF analyses were performed on both systems. RMSF analyses provided different fluctuation profiles, especially for the regions 20–37 and 96–104 (Figure 3). The regions 20–37 and 97–104 were organized in an alpha–alpha hairpin and a random loop, respectively, interacting between them in the starting structure of the MD simulation. Interestingly, in SAA1.2, the alpha–alpha hairpin (20–37) and random loop (97–104) regions were highly stable along the entire MD run exhibiting several hydrophobic and polar interactions. By contrast, SAA1.1 first showed a destabilization of the alpha–alpha hairpin region (20–37), followed by a lack of all interactions with the C-ter region (97–104), causing its high structural instability (Figure 3).

### 3.3. Principal Component (PC), Conformational, and Intramolecular Interaction Network Analyses

**Figure 4:**
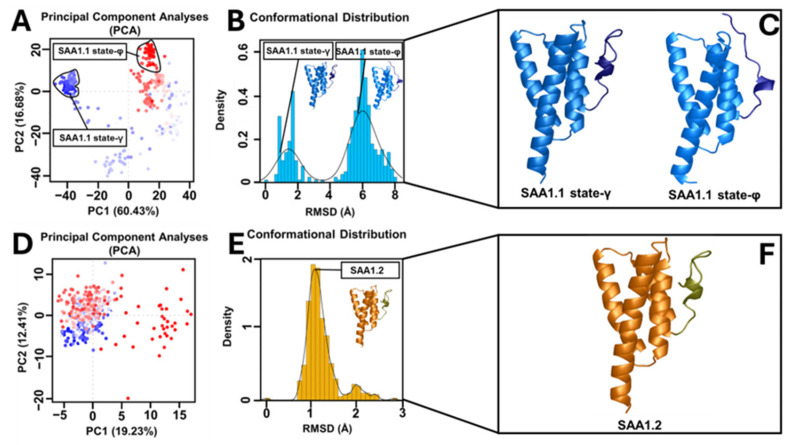
Principal component, conformational, and RMSD distribution analyses. PCAs of (**A**) SAA1.1 and (**D**) SAA1.2. The black and red points represent PC1 and PC2, respectively. The conformational distribution based on the RMSD values was reported for (**B**) SAA1.1 and (**E**) SAA1.2. Conformational analyses of (**C**) SAA1.1 and (**F**) SAA1.2. The C-ter region of both systems is reported in a darker color. Only SAA1.1 presented two different conformational states.

To dissect the C-ter region features of the systems, PCA studies were performed. The PCAs of the MD trajectories of each system considering the C-ter region revealed that most of the conformations of SAA1.2 were confined within a single subspace, which differed from the SAA1.1, which showed two different subspaces (Figure 4A–D). RMSD distribution analyses (Figure 4B–E) were performed for each system and confirmed PCAs, showing a clear presence of one only conformational population for SAA1.2, where the C-ter region was close to the alpha–alpha hairpin region (state-γ), while for SAA1.1 two main conformational states were observed: state-γ and a second state where the C-ter region was far from the alpha–alpha hairpin region (state-φ) (Figure 4C–F).

**Figure 5:**
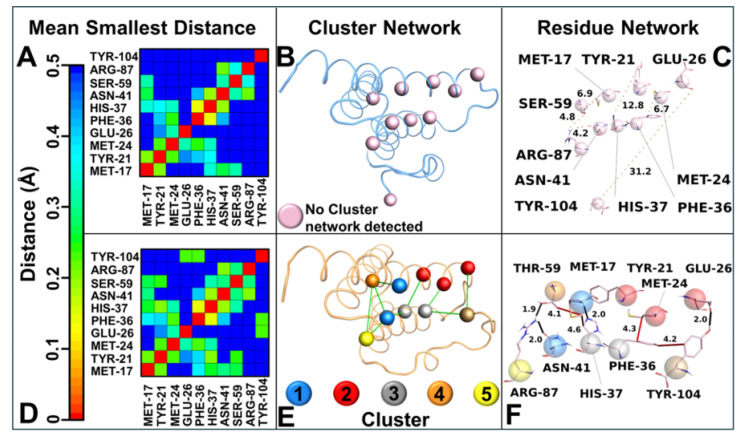
Cluster interaction network details. Distance matrices consist of the smallest distance between residue pairs for (**A**) SAA1.1 and (**D**) SAA1.2. Correlation cluster network analysis for (**B**) SAA1.1 (blue ribbon) and (**E**) SAA1.2 (pink ribbon) SAA1.1. In pink and colored spheres, the C-alpha belonged to clusters detected by Bio3D. In SAA1.1, no cluster was detected, while in SAA1.2 a correlation cluster network (green line) was retrieved among five clusters. To clarify the picture, SAA1.1 has the same clusters as SAA1.2 to allow a comparison; however, no correlation was present for SAA1.1. Residue network for SAA1.1 (**C**) and SAA1.2 (**F**). The interaction residues were reported in pink sticks, while the C-alpha was represented in transparency with the same colors as the correlation cluster network. The black and red lines represent hydrogen bonds and hydrophobic interactions, respectively, while the yellow dotted lines represent no interaction. The number on the line represents the bond distance in Å.

Intramolecular interaction networks for both systems were evaluated by applying different analyses. Firstly, the intramolecular contact map of each SAA1 variant was evaluated with GROMACS 2019.3 (with the gmx mdmat function). The contact map showed notable differences, highlighting the ability of SAA1.2 to form a wider interaction network as well as shorter distances among the residues than SAA1.1 (Figure 5A–D). Further investigations were conducted by using the BIO3D library implemented in Rstudio to evaluate the correlation network analysis (CNA) of the MD trajectory of SAA1 variants. The CNA results did not detect any network for SAA1.1 (Figure 5B); however, in SAA1.2 a significant cluster interaction network was detected. In-depth CNA analyses showed the presence of five clusters correlated among them (Figure 5E). In the SAA1.1 cluster interaction network, no interaction was observed among the clusters (Figure 5C), while in SAA1.2 a fine and intricate hydrophobic and polar interaction network was formed, involving residues from the N-ter region to the C-ter region (Figure 5F). Interestingly, several interactions involved in the stability of the SAA1.2 were observed; of particular interest, the Thr-59 formed a strong H-bond with Arg-87 and a hydrophobic interaction with Met-17 for the entire MD run.

## 4. Discussion

The investigation of structural conformations and dynamic stability of the SAA1 protein variants (SAA1.1 and SAA1.2) offers profound insights into their distinct molecular behaviors.

While SAA proteins, particularly SAA1, have been studied in other amyloid-related diseases [17], our study uniquely links the SAA1.1 isoform to AKU severity through computational modeling, providing new insights into its potential role as a biomarker.

Our study integrates clinomics and molecular dynamics (MD) simulations to predict molecular behaviors and disease outcomes. Clinomics, which combines clinical data with high-throughput “-omics” technologies, provides a framework for understanding disease pathways and identifying biomarkers by linking clinical phenotypes to molecular profiles. This approach enabled us to establish a novel connection between the SAA1.1 allelic variant and heightened disease severity in alkaptonuria (AKU), moving beyond mere observation to predictive insights. By identifying the SAA1.1 isoform’s destabilizing effects, we demonstrated how MD simulations can predict the structural instability of proteins, revealing conformational shifts that drive amyloid fibril formation and inflammation.

However, the inherent ultra-rarity of AKU poses significant challenges to achieving conventional statistical significance, requiring an analytical approach that emphasizes descriptive and exploratory methods suited for small sample sizes. Figure 1 illustrates the range, distribution, and variability of key biochemical markers across allelic groups. Rather than serving as a basis for definitive statistical conclusions, it highlights observable patterns and trends that may hold biological and clinical relevance. These trends, such as potential links between SAA1 variants and inflammatory markers, are further supported by molecular dynamics analysis, offering a multidimensional perspective on the data and guiding hypothesis generation. This approach aligns with best practices in rare disease research, where descriptive and exploratory analyses often advance knowledge despite sample size constraints. By integrating visualization with molecular modeling, this study maximizes the utility of the available data while addressing their limitations, providing a strong foundation for future investigations.

MD simulations, as applied in our study, offer a mechanistic understanding of these molecular events, surpassing experimental validation by predicting how genetic variations impact protein behavior. These simulations enabled us to foresee how structural changes in the SAA1.1 variant lead to pathological transitions, thus providing a predictive model for disease severity. This predictive framework opens new avenues for therapeutic strategies, such as stabilizing agents to prevent amyloidogenesis or mitigate inflammatory responses.

Together, clinomics and MD simulations enhance our ability to predict the outcomes of genetic variations, offering a dual approach to disease progression and therapeutic development. By capturing transient states and revealing molecular instability that is difficult to observe experimentally, these methods provide valuable insights that can guide precision medicine strategies tailored to individual genetic profiles.

These implications are particularly noteworthy for alkaptonuria. AKU is a rare disorder characterized by the accumulation of homogentisic acid and associated complications, including tissue degeneration and amyloidosis, identifying SAA1.1 as a potentially destabilizing variant that offers a novel biomarker for predicting disease severity. This insight provides a transformative approach to AKU management by enabling the early diagnosis and severity assessment of secondary amyloidosis and exemplifying how structural biology can inform early interventions, potentially mitigating irreversible damage associated with disease progression. Genetic stratification of patients through genotype analysis can guide personalized interventions, with monoclonal antibodies targeting inflammatory mediators, such as tocilizumab [15], presenting a potential prevention/therapeutic option. Furthermore, linking allelic variants to disease progression establishes a foundation for precision medicine strategies tailored to the molecular profiles of individual patients.

Beyond AKU, this work has broader relevance for amyloid-related diseases, where protein misfolding and aggregation play central roles in pathology. The innovative use of molecular dynamics simulations and interaction network analyses sets a new standard for exploring protein variant stability. This approach can be applied to identify critical molecular markers and therapeutic targets in other conditions where amyloidogenesis contributes to disease, such as Alzheimer’s or systemic amyloidosis.

By employing a comprehensive 1 μs classical molecular dynamics (cMD) simulation, coupled with analyses such as RMSD, RMSF, principal component analysis (PCA), and intramolecular interaction networks, this study delineates the fundamental factors contributing to the divergent structural stabilities and interaction profiles of these variants. The implications of these findings extend to understanding the functional disparities between the variants and providing a molecular basis for their differential roles in pathological and physiological contexts.

The root mean square deviation (RMSD) results provide a clear comparative view of the structural stability between the SAA1.1 and SAA1.2 isoforms over the molecular dynamics (MD) trajectory. The RMSD measures the average deviation of atomic positions from a reference structure and serves as a reliable indicator of overall structural stability. For SAA1.2, RMSD values remained consistently low throughout the simulation, reflecting robust structural integrity. This stability suggests that SAA1.2 retains its native conformation under simulated conditions, with minimal deviation over time. Such behavior is characteristic of a well-folded protein with a stable intramolecular interaction network.

In contrast, the SAA1.1 isoform displayed a markedly different RMSD profile. While initially stable for the first 700 nanoseconds (ns) of the simulation, SAA1.1 underwent a significant conformational shift, as evidenced by a sharp increase in RMSD to approximately 6 Å. This sudden and pronounced deviation signifies a destabilizing event, likely linked to the intrinsic structural properties of the isoform. The observed RMSD spike underscores a fundamental loss of stability, prompting further investigation into the specific molecular features responsible for this destabilization.

To isolate the source of this structural instability, additional RMSD analyses were conducted, excluding the C-terminal (C-ter) region from the calculations. Interestingly, when the C-ter region was excluded, both SAA1.1 and SAA1.2 exhibited comparable RMSD values of approximately 1.5 Å, indicating that the backbone dynamics of the core region (excluding the C-ter) are similar for both isoforms. This finding highlights the pivotal role of the C-ter region in modulating the structural integrity of SAA1.1 and implicates it as the primary driver of the observed destabilization.

Building on these findings, root mean square fluctuation (RMSF) analyses were employed to gain residue-specific insights into the flexibility and dynamic behavior of the protein. RMSF measures the average fluctuation of each residue around its mean position, providing a granular view of local flexibility. The analysis revealed significant differences in the dynamic profiles of the two isoforms. Specifically, two regions of interest—the alpha–alpha hairpin (residues 20–37) and the random loop (residues 96–104)—exhibited strikingly distinct behavior between the isoforms.

In SAA1.2, these regions demonstrated high stability throughout the simulation. The alpha–alpha hairpin and the random loop remained in close proximity, maintaining critical hydrophobic and polar interactions. These interactions serve to anchor the C-ter region to the rest of the protein, ensuring the structural cohesion of the molecule. This stability likely contributes to the robust conformational integrity observed in the SAA1.2 isoform.

Conversely, in SAA1.1, the alpha–alpha hairpin exhibited destabilization, characterized by increased fluctuations and disrupted interactions with the C-ter region. This loss of stabilizing interactions between the alpha–alpha hairpin and the C-ter led to a cascading destabilization of the entire C-ter region. The decoupling of these domains not only resulted in localized instability but also amplified the overall structural deviation of the protein. Such behavior is indicative of an inherent vulnerability in the SAA1.1 isoform, driven by its unique sequence and structural properties.

These findings underscore the critical interplay between the alpha–alpha hairpin and the C-ter region in maintaining the structural integrity of SAA proteins. In SAA1.2, the cohesive interaction network between these domains fortifies the protein against destabilizing forces, while in SAA1.1, the breakdown of this network results in significant conformational instability. This comparative analysis highlights the molecular basis for the differential stability of the two isoforms, offering valuable insights into their distinct functional and pathological roles.

Principal component analysis (PCA) of the MD trajectories revealed divergent conformational landscapes for the two variants. SAA1.2 confined most of its conformations within a single subspace, corresponding to a stable state where the C-ter region is proximate to the alpha–alpha hairpin. In stark contrast, SAA1.1 exhibited two distinct subspaces: the stable state-γ and a secondary state (state-φ), characterized by the C-ter region’s departure from the alpha–alpha hairpin. The dual-state behavior of SAA1.1 aligns with its heightened structural instability and susceptibility to conformational shifts. Intramolecular interaction analysis further elucidated the mechanistic underpinnings of variant-specific stability. The contact map analysis highlighted that SAA1.2 fosters a broader interaction network with shorter residue–residue distances, whereas SAA1.1 exhibits a more fragmented interaction profile lacking important interactions. These observations were corroborated by correlation network analysis (CNA), which identified an intricate hydrophobic and polar interaction network in SAA1.2, encompassing residues from the N-terminal (N-ter) to the C-ter region.

The intricate network of intramolecular interactions revealed in this study sheds light on a critical determinant of SAA1 variant stability—Thr-59 in SAA1.2. This residue, solely present in SAA 1.2 among all variants, emerges as a pivotal stabilizing force, enabling SAA1.2 to maintain structural integrity and foster effective interplay between key structural domains. By contrast, the SAA1.1 variant lacks this stabilizing feature, as it contains serine (Ser-59) in place of threonine, a substitution with profound ramifications for its structural and dynamic behavior.

In SAA1.2, Thr-59 acts as a molecular linchpin, forming a strong hydrogen bond with Arg-87 and a consistent hydrophobic interaction with Met-17. This dual interaction anchors the alpha–alpha hairpin (residues 20–37) to the random loop (residues 96–104), reinforcing their spatial proximity and promoting structural stability in the C-terminal (C-ter) region. This interplay creates a robust molecular framework, ensuring that the C-ter region remains in the γ-conformational state, where it is closely associated with the alpha-alpha hairpin. This configuration stabilizes the overall protein structure and optimizes the hydrophobic and polar interaction network critical for the biological function of SAA1.2. Conversely, SAA1.1 is deprived of this stabilizing interaction due to the substitution of Thr-59 with Ser-59. Unlike threonine, serine lacks a methyl group, a structural feature indispensable for hydrophobic interactions with Met-17. The absence of this methyl group eliminates the stabilizing hydrophobic contact and weakens the interaction with Arg-87. Consequently, the interplay between the alpha–alpha hairpin and random loop is significantly impaired in SAA1.1, causing a breakdown in the cohesive interaction network responsible for the C-ter stability. This disruption is a primary factor behind the conformational heterogeneity observed in SAA1.1, as evidenced by its tendency to alternate between the γ-state and a destabilized φ-state, where the C-ter region diverges from the alpha–alpha hairpin. The role of Thr-59 extends beyond local interactions, influencing the broader structural dynamics of SAA1.2. By stabilizing the alpha–alpha hairpin and random loop, Thr-59 ensures the preservation of hydrophobic and polar networks that span from the N-terminal (N-ter) to the C-ter region. This interconnected network promotes uniform conformational dynamics, as demonstrated by PCA results showing that SAA1.2 conformations are confined to a single stable subspace. This structural coherence is absent in SAA1.1, where the lack of Thr-59 contributes to the fragmentation of the interaction network, destabilizing the alpha–alpha hairpin and weakening its coordination with the C-ter region.

The absence of Thr-59 in SAA1.1 not only undermines the integrity of the alpha–alpha hairpin but also disrupts its role as a structural mediator. The resulting decoupling between the hairpin and the random loop precipitates a cascade of destabilizing effects, leaving the C-ter region vulnerable to conformational fluctuations. This is reflected in the PCA and RMSD analyses, where SAA1.1 exhibits a dual conformational state and increased structural deviation, hallmarks of reduced stability. Thr-59 represents a cornerstone of SAA1.2 stability, acting as a molecular nexus that links key structural domains and preserves the cohesiveness of the protein. Its unique ability to engage in both hydrogen bonding and hydrophobic interactions distinguishes it as an irreplaceable element of the SAA1.2 interaction network. The absence of Thr-59 in SAA1.1, replaced by the less versatile Ser-59, underscores the delicate balance required for maintaining structural stability. This single-residue substitution exemplifies the profound impact of subtle amino acid variations on protein dynamics and highlights the vulnerability of SAA1.1 to destabilizing perturbations. The findings underscore the centrality of Thr-59 in dictating not only the stability of SAA1.2 but also its potential functional roles. The preservation of a cohesive interaction network and a stable C-ter region is likely to confer functional advantages to SAA1.2, enhancing its ability to interact with ligands or participate in multimeric assemblies. By contrast, the compromised structural stability of SAA1.1 may predispose it to pathological transitions, such as misfolding or aggregation, which are often implicated in amyloid-related diseases.

Briefly, Thr-59 serves as a molecular cornerstone in SAA1.2, ensuring the stable interplay between the alpha–alpha hairpin and random loop regions and safeguarding the structural integrity of the C-ter region. The absence of this critical residue in SAA1.1 underscores the profound implications of single-residue substitutions, shedding light on the mechanistic underpinnings of variant-specific dynamics and their broader functional consequences. This finding accentuates the power of molecular simulations and interaction network analyses to unravel intricate structural phenomena, offering a compelling narrative for the centrality of Thr-59 in maintaining protein stability and function. This comprehensive analysis delineates the molecular determinants underlying the differential stability of SAA1 variants. The stable structural dynamics of SAA1.2 are driven by a synergistic interplay of robust intramolecular interactions, conformational uniformity, and network connectivity, positioning it as a structurally superior variant. In contrast, the pronounced destabilization in SAA1.1 arises from localized disruptions, particularly within the alpha–alpha hairpin and its interaction with the C-ter region.

These findings hold significant implications for understanding the functional roles of SAA1 variants in AKU and other amyloidosis pathological contexts where protein misfolding or instability plays a role. The ability of SAA1.2 to sustain a stable conformation and maintain an intricate interaction network may confer functional advantages, potentially influencing its interactions with ligands or other biomolecules. Meanwhile, the instability of SAA1.1 could predispose it to pathogenic transitions, such as aggregation or misfolding, relevant to amyloid diseases.

## 5. Conclusions

Serum amyloid A (SAA) is a well-characterized acute-phase protein predominantly produced by hepatocytes in response to pro-inflammatory cytokines such as IL-6, IL-1β, and TNF-α during systemic inflammation.

Its physiological role includes modulating immune responses, promoting chemotaxis, and participating in high-density lipoprotein metabolism during acute and chronic inflammatory states. 

In the context of AKU, chronic inflammation arising from ochronotic pigment deposits—formed by homogentisic acid (HGA) polymerization within connective tissues—leads to significant local and systemic upregulation of SAA. This aligns with its known function as a biomarker of persistent inflammation associated with tissue damage. However, as previous studies and findings in the present manuscript demonstrate, the elevated levels of SAA observed in AKU patients cannot be solely attributed to ochronosis-related inflammation. The homozygosity of the SAA1.1 allele plays a distinct and pivotal role in exacerbating the inflammatory response. Structural instability inherent to the SAA1.1 isoform renders it more prone to amyloidogenic transformations, characterized by the misfolding and aggregation of the protein into pathological β-sheet-rich fibrils.

This isoform-specific susceptibility is directly linked to the heightened and accelerated inflammatory progression observed in individuals homozygous for SAA1.1, in contrast to carriers of other polymorphic variants such as SAA1.2.

This manuscript provides compelling evidence from molecular dynamics simulations showing that the SAA1.1 isoform exhibits significant conformational destabilization, particularly in the C-terminal region. This structural instability facilitates the generation of amyloidogenic fragments that exacerbate inflammation by activating immune cells through pattern recognition receptors, such as Toll-like receptors (TLRs). These pathways amplify the production of pro-inflammatory cytokines, creating a feedback loop that further elevates SAA levels and promotes secondary amyloidosis.

This finding is consistent with studies identifying the SAA1.1 isoform as the predominant variant in amyloid deposits across multiple inflammatory conditions. The polymorphism’s unique sequence features, including specific residue substitutions, enhance its susceptibility to degradation and amyloid fibril formation, distinguishing it from other SAA1 isoforms.

This study represents a significant advancement in understanding the molecular mechanisms underlying alkaptonuria (AKU) and related amyloid diseases, highlighting the potential of computational approaches to inform biomarker discovery and therapeutic development. By focusing on structural and dynamic differences between SAA1 protein variants derived from AKU patients, this work bridges fundamental molecular biology with translational medicine. The findings emphasize how single-residue variations can drastically influence protein stability, offering deep insights into the molecular underpinnings of disease progression. The study’s implications for alkaptonuria (AKU) are significant, as AKU is a rare disorder marked by homogentisic acid accumulation, tissue degeneration, and amyloidosis. Identifying SAA1.1 as a destabilizing variant provides a novel biomarker for predicting disease severity, enabling the early diagnosis of secondary amyloidosis and assessment of progression. This insight offers a transformative approach to AKU management, guiding personalized interventions, such as monoclonal antibodies like tocilizumab [15]. The data presented here support the role of SAA1.1 as a novel biomarker for AKU. Our findings provide strong mechanistic insights into how the destabilizing nature of SAA1.1 contributes to disease progression, laying the groundwork for potential therapeutic interventions. However, linking allelic variants to disease progression supports precision medicine strategies tailored to individual molecular profiles. Beyond AKU, this work holds broader relevance for amyloid-related diseases, where protein misfolding and aggregation drive pathology.

This integrative methodology not only emphasizes the power of combining clinomics with MD simulations but also addresses a critical gap in AKU research by linking allelic variants to specific molecular mechanisms of disease progression. Its relevance extends beyond AKU, providing a paradigm for using computational tools to transform our understanding and treatment of protein misfolding diseases. The study’s impact is poised to resonate across scientific and medical communities, enhancing our ability to understand and treat the profound impact that even single-point allelic variations exert on protein structure, stability, and their critical role in the progression of diseases. By establishing these allelic differences as biomarkers to disease progression, our work underscores the importance of genetic profiling in understanding and managing complex diseases.

In summary, while ochronotic pigment deposits within connective tissues undoubtedly trigger a pro-inflammatory response and elevate SAA levels as part of the acute-phase reaction, the presence of the SAA1.1 allele in a homozygous state significantly amplifies this effect. The allele-specific structural instability of SAA1.1 accelerates amyloidogenic processes, driving a more rapid and severe inflammatory progression in affected individuals. This underscores the importance of genotypic screening for SAA1.1 as a predictive biomarker for inflammation severity and amyloid-related complications in AKU and other chronic inflammatory diseases. Future therapeutic strategies targeting the stabilization of SAA1.1 or its amyloidogenic intermediates may hold promise for mitigating disease progression.

## Data Availability

No new data were created.
